# Circulating levels of sclerostin are associated with cardiovascular mortality

**DOI:** 10.1371/journal.pone.0199504

**Published:** 2018-06-21

**Authors:** Cristina Novo-Rodríguez, Beatriz García-Fontana, Juan De Dios Luna-Del Castillo, Francisco Andújar-Vera, Verónica Ávila-Rubio, Cristina García-Fontana, Sonia Morales-Santana, Pedro Rozas-Moreno, Manuel Muñoz-Torres

**Affiliations:** 1 Endocrinology and Nutrition Unit, Hospital Universitario San Cecilio. Instituto de Investigación Biosanitaria de Granada (Ibs.GRANADA). Av. de la Ilustración, s/n, Granada, Spain; 2 CIBERFES. Instituto de Salud Carlos III. Carretera de Majadahonda—Pozuelo, Km. 2.200, Majadahonda, Madrid, Spain; 3 Department of Biostatistical, University of Granada. Av. de la Investigación, Granada, Spain; 4 Proteomic Research Service, Fundación para la Investigación Biosanitaria de Andalucía Oriental- Alejandro Otero. Instituto de Investigación Biosanitaria de Granada (Ibs.GRANADA). Av. Doctor Olóriz 16, Granada, Spain; 5 Endocrinology Division, Hospital General de Ciudad Real. C/ Obispo Rafael Torija, s/n, Ciudad Real, Spain; 6 Department of Medicine. University of Granada. Av. de la Investigación, Granada, Spain; The University of Tokyo, JAPAN

## Abstract

Cardiovascular diseases are a health problem throughout the world, especially in people with diabetes. The identification of cardiovascular disease biomarkers can improve risk stratification. Sclerostin is a modulator of the Wnt/β-catenin signalling pathway in different tissues, and it has recently been linked to vascular biology. The current study aimed to evaluate the relationship between circulating sclerostin levels and cardiovascular and non-cardiovascular mortality in individuals with and without type 2 diabetes. We followed up a cohort of 130 participants (mean age 56.8 years; 48.5% females; 75 with type 2 diabetes; 46 with prevalent cardiovascular disease) in which serum sclerostin levels were measured at the baseline. Time to death (both of cardiovascular and non-cardiovascular causes) was assessed to establish the relationship between sclerostin and mortality. We found that serum sclerostin concentrations were significantly higher in patients with prevalent cardiovascular disease (*p*<0.001), and independently associated with cardiovascular mortality (*p* = 0.008), showing sclerostin to be a stronger predictor of mortality than other classical risk factors (area under the curve = 0.849 *vs* 0.823). The survival analysis showed that an increase of 10 pmol/L in the serum sclerostin level resulted in a 31% increase in cardiovascular mortality. However, no significant association was observed between sclerostin levels and non-cardiovascular mortality (*p* = 0.346).

From these results, we conclude that high sclerostin levels are related to mortality due to cardiovascular causes. The clinical implication of these findings is based on the possible use of serum sclerostin as a new biomarker of cardiovascular mortality risk in order to establish preventive strategies.

## Introduction

Cardiovascular disease (CVD) is currently a major cause of death worldwide, contributing to 42% of deaths among women and 38% among men below the age of 75 years, in Europe [1]. Atherosclerosis is the main process that leads to the development of macrovascular complications including CVD. Several risk factors lead to the continuous recruitment of inflammatory cells, proliferation of vascular smooth muscle cells (VSMCs), cholesterol accumulation and vascular calcification, which determine the growth of atherosclerotic lesions [2]. Type 2 diabetes (T2D) is considered an independent risk factor for CVD, in both men and women [3] which is leading to 70–80% of all deaths among diabetic patients [4,5]. The development of atherosclerotic CVD has a complex and multifactorial origin that is determined by several classical cardiovascular risk factors and others that are still not understood in-depth. Therefore, finding the predictor molecules of cardiovascular mortality could provide an effective strategy to make a better identification of patients with higher cardiovascular risk.

Sclerostin is a glycoprotein, secreted primarily, but not exclusively, by osteocytes. It is one of the main regulators of the canonical Wnt/β-catenin signalling pathway, and it mainly acts as an inhibitor of bone formation [6,7]. Since sclerostin has been associated with several bone metabolism disorders, its role in the inhibition of osteoblastogenesis has opened a new area for the development of therapeutic strategies for metabolic bone diseases. However, there is increasing evidence on the extraskeletal functions of sclerostin, pointing to its role in various vascular disorders. Recently published data have shown that, under calcifying conditions, VSMCs are capable of producing a phenotypic transition to osteoblast-like cells which are able to express the typical bone markers, including sclerostin [8]. Some studies have reported increased levels of sclerostin in patients with calcification of the vascular tissue [9,10] as well as the involvement of sclerostin in several disorders related to vascular calcification processes [11,12]. However, the mechanism by which sclerostin can influence the calcification process is controversial [13–17]; some of them suggest that sclerostin has a protective role [18] while others suggest the opposite [19].

On the other hand, few studies have focused on the usefulness of sclerostin as a predictor of mortality. Recently published data are contradictory, in terms of the relationship between sclerostin and mortality in subjects with chronic kidney disease [19–23]. Moreover, it is not clear if high circulating levels of sclerostin are a risk factor for mortality in the case of T2D patients and those without diabetes.

In this context, our aim was to assess the usefulness of sclerostin levels as a predictor of mortality due to cardiovascular and non-cardiovascular causes in a mixed population including individuals with and without T2D.

## Material and methods

### Study population and ethics statement

This longitudinal observational pilot study included 130 participants with a mean age of 58.8 years, and a similar percentage of men and women. Seventy five participants had T2D while 55 were non-diabetic. Diabetes mellitus was diagnosed according to American Diabetes Association criteria from 2005. From January 2006 to March 2007, we consecutively recruited patients who had been referred to our outpatient clinic from primary care centres for the treatment of diabetes. The non-diabetic participants were consecutively recruited from the general community in the same period.

Participants were classified into two groups, according to the presence of prevalent CVD. Inclusion criteria for CVD were cerebrovascular disease (ischemic stroke or transient ischemic attack), coronary heart disease (previous myocardial infarction, diagnosed unstable angina, coronary revascularization surgery or percutaneous coronary interventions), or ischemic peripheral arterial disease.

**Fig 1:**
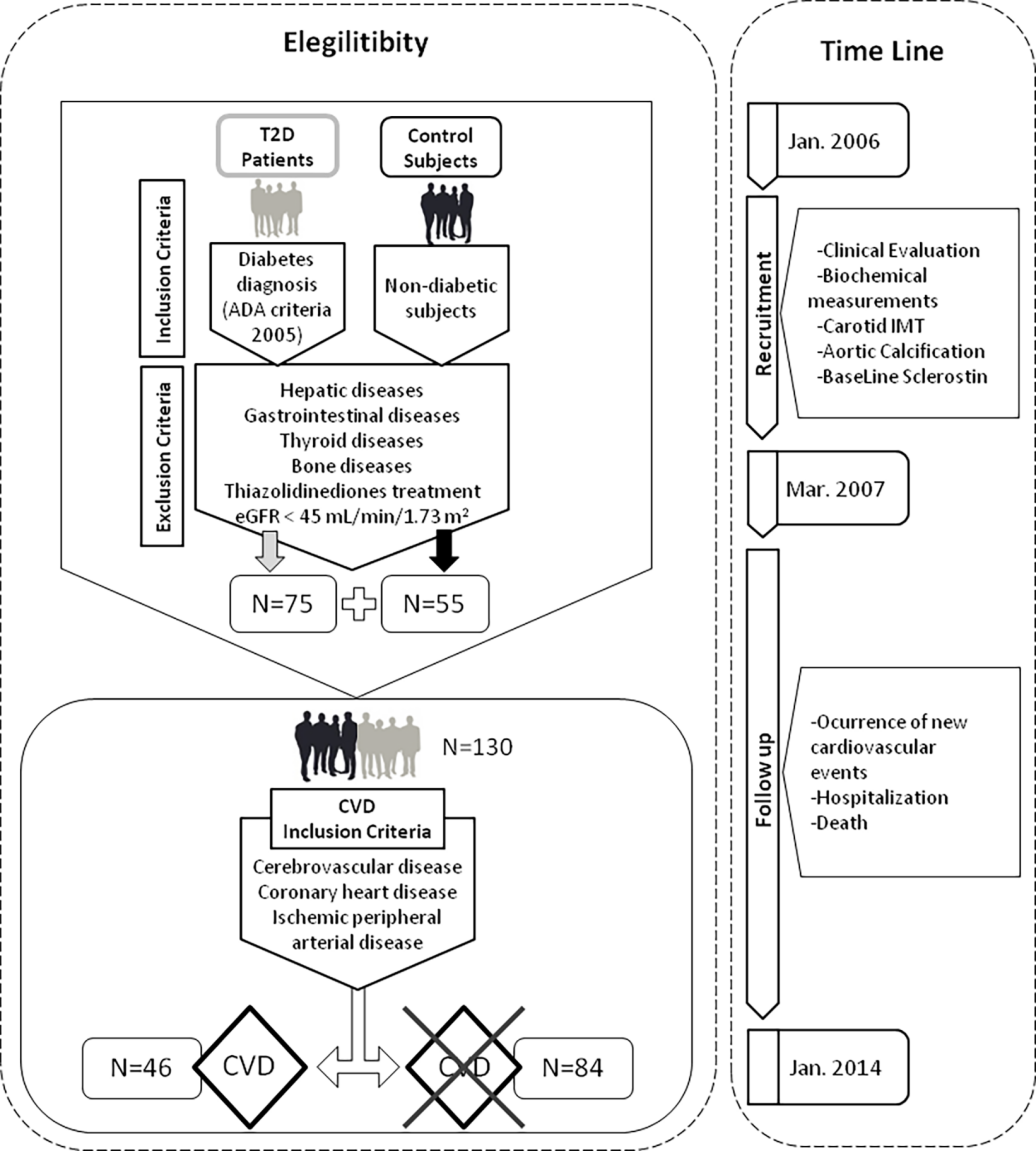
Flowchart indicating the study design, and the inclusion and exclusion criteria for recruitment.

All included patients were followed-up for time-to-event analysis until January 2014. We analysed the occurrence of new cardiovascular events and death (cardiovascular or non-cardiovascular). Information was obtained from San Cecilio University Hospital’s records. All participants were Caucasians and ambulatory, and did not have hepatic, gastrointestinal, thyroid or bone diseases. The T2D patients were undergoing treatment with antidiabetic drugs, including metformin, sulfonylureas, insulin or a combination of these drugs. Patients with an estimated glomerular filtration rate (eGFR) below 45 mL/min/1.73 m^2^ and those treated with thiazolidinediones due to the influence of this drug on bone metabolism and on the risk of cardiovascular events were excluded (Fig 1).

The study was conducted with the approval of the ethics committee of the San Cecilio Hospital of Granada and conformed to the principles of the World Medical Association Declaration of Helsinki (Project ID: PI 0514–2012. Research Ethics Committee of Granada Center (CEI-Granada) at 26 November 2012). Written informed consent was obtained from all participants.

### Biochemical measurements

Serum sclerostin was measured using a commercially available ELISA kit (Biomedica, Vienna, Austria), according to the manufacturer’s instructions. In our laboratory, we assay duplicates for all values. Precision testing was performed by the determination of intra-assay and inter-assay variations. Two samples of known concentrations were tested six times for the assessment of intra-assay variability, which yielded a result of 4%, while two samples of known concentrations were tested in three assays from two different operators to the assess inter-assay variability, yielding a result of 3%. Sclerostin measurements are reported in pmol/L, and the lower limit of detection in our study was 10 pmol/L.

Clinical evaluation, biochemical measurements, carotid intima-media thickness (IMT) and aortic calcification measurements were evaluated at the baseline, following the procedures described by Morales-Santana *et al* [10]. The eGFR was calculated using the Chronic Kidney Disease Epidemiology Collaboration equation [24].

### Statistical analysis

Data were expressed as means ± SD for variables normally distributed and as median with the interquartile range (IQR) for variables not normally distributed. Data for categorical variables were presented as percentages. A Shapiro-Wilks test was used to test the normality of distribution of the continuous variables. The mean values between groups were compared using the unpaired Student t test for continuous and normally distributed variables. The Mann-Whitney U test was used to compare variables not normally distributed. The χ^2^ test was used to compare categorical variables between groups. Associations between continuous variables were described by Pearson’s or Spearman’s correlation coefficients.

A multiple lineal regression model was performed to determine the variables independently associated with sclerostin, including the quantitative and qualitative variables linked in the bivariate analysis, and other variables biologically associated to sclerostin.

To identify sclerostin as an independent predictor of mortality, a multiple logistic regression model was performed, including mortality as a dependent variable. The independent variables included in the model, in addition to sclerostin, were the established risk factors of mortality (age, sex, hypertension, tobacco use, prevalent cardiovascular events, pathological intima-media thickness (pIMT), as well as the presence and duration of diabetes) and eGFR. The usefulness of serum sclerostin as a marker of mortality risk was analysed using a receiver operating characteristic (ROC) curve. The area under the curve (AUC) indicates the probability to predict an event. AUC values greater than 0.75 indicate a good predictive performances.

Since cardiovascular events are competitors for mortality due to the presentation of each event prevents the presentation of the others, a competing risk regression is the most appropriate model to assess the risk of mortality [25,26]. We have used Fine and Gray model, because it considers both death and the presence of cardiovascular events as competing events adjusting by exposure time [27]. However, we also included the results obtained through the Cox and Kaplan-Meier methodology, in order to obtain comparable results with other studies that used survival analysis without competing risk.

To facilitate the interpretation of the data, the values of sclerostin were expressed as pmol/Lx10 units. A *p* value ≤ 0.05 was considered significant (two-tailed). All analyses were performed using STATA 14.2 and those for competitive risks were performed using the stcrreg program (STATA).

## Results

### Baseline characteristics of the study population

The whole sample was divided in two groups, according to the presence of prevalent CVD. The clinical and demographic characteristics of the study groups are summarized in Table 1. Both groups differed in cardiovascular risk factors, CVD-defining parameters and CVD-subrogated markers. As expected, the group with CVD showed higher levels of glycated haemoglobin (HbA1c) and fasting plasma glucose (FPG), as well as higher cardiovascular and non-cardiovascular mortality than those without CVD. No differences in the eGFR were found between the groups.

**Table 1:** Anthropometric and biochemical parameters of the study participants according to the cardiovascular disease status.

	CVD group (n = 46)	Non-CVD group (n = 84)	*p* value
Age (years)	60.0 (56.0–64.0)	56.0 (51.0–75.0)	0.001
Male/female (n)	30/16	37/47	0.020
**Measurements**			
BMI (kg/m^2^)	30.5 (27.8–33.6)	27.8 (25.4–33.1)	0.089
Waist circumference (cm)	107.0 ± 10.7	99.7 ± 12.6	0.001
SBP (mm Hg)	135 ± 20	130 (110–140)	0.081
DBP (mm Hg)	77 ± 12	80 (70–90)	0.170
IMT (mm)	0.86 ± 0.15	0.67 (0.60–0.75)	< 0.001
**Medical history**			
T2D (%)	93.8	37.6	< 0.001
Duration of diabetes (years)	14.1 ± 7.7	12.45 ± 7.4	0.350
Hypertension (%)	83.3	55.3	0.001
Dyslipidaemia (%)	97.9	74.1	0.001
Smoker (%)	12.5	18.8	0.340
Sedentarism (%)	56.3	42.4	0.120
Coronary heart disease (%)	62.5	0	< 0.001
Cerebrovascular disease (%)	35.4	0	< 0.001
Peripheral artery disease (%)	23.8	0	< 0.001
Carotid plaques (%)	33.3	6.2	< 0.001
Aortic calcifications (%)	43.2	9.5	< 0.001
pIMT (%)	64.6	21.2	< 0.001
Total mortality (%)	30.4	4.9	< 0.001
CVD-related mortality (%)	19.6	3.7	0.003
**Serum parameters**			
FPG (mmol/L)	8.44 (6.65–11.57)	5.27(4.77–8.27)	< 0.001
HbA1c (mmol/mol/ %)	55.19 (43.71–68.30)/ (7.5)	29.50 (24.04–53.55)/ (5.2)	< 0.001
Triglyceride (mmol/L)	1.31(0.97–1.88)	1.15 (0.81–1.58)	0.320
HDL-c (mmol/L)	1.19 (0.94–1.34)	1.50 ± 0.39	< 0.001
LDL-c (mmol/L)	2.33 ± 0.88	3.42 ± 0.80	< 0.001
eGFR (mL/min/1.73 m^2^)	82.20 ± 18.31	88.40 (72.50–100.00)	0.144
Sclerostin (pmol/L)	50.93 (34.28–77.35)	40.45 (31.62–50.59)	0.001

BMI: body mass index; CVD, cardiovascular disease; DBP: diastolic blood pressure; CKD: chronic kidney disease; FPG: fasting plasma glucose; eGFR: estimated glomerular filtration rate; HbA1c: glycated haemoglobin; T2D: type 2 diabetes; HDL-c: high-density lipoprotein cholesterol; IMT: intima-media thickness; LDL-c: low-density lipoprotein cholesterol; pIMT: pathological intima-media thickness. SBP: systolic blood pressure. Data for continuous and normally distributed variables are presented as mean ± SD. Data for continuous variables not normally distributed, are presented as median followed by IQR in brackets. Data for categorical variables are presented as percentages.

### Determinants of sclerostin level

**Fig 2:**
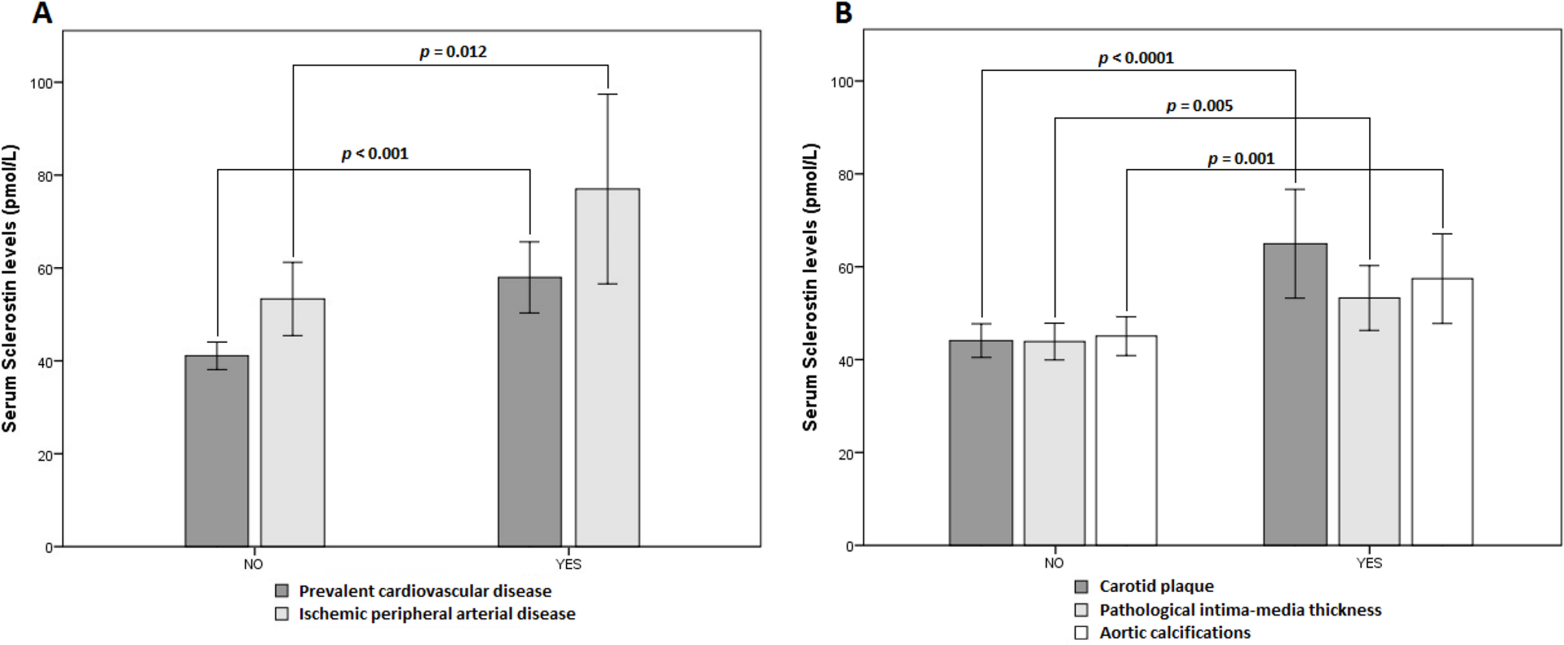
Serum sclerostin levels in the study participants according to the presence of cardiovascular parameters. (A) Serum sclerostin levels according to the presence of prevalent CVD in the entire cohort and according to the presence of ischemic peripheral arterial disease in the CVD group; (B) Serum sclerostin levels according to the presence of abnormal subrogated markers of CVD (carotid plaque, pIMT and aortic calcifications) in the entire cohort. Data are means ± 95% CI. Significant differences between group regions are indicated by a bar with the *p*-value given above. CVD: cardiovascular disease; pIMT: pathological intima media thickness; CI: confidence interval.

We found increased levels of serum sclerostin in patients with prevalent CVD, compared to those without prevalent CVD (57.96 ± 25.75 *vs* 43.61 ± 18.79 pmol/L, *p*<0.001). In the group with prevalent CVD, participants with ischemic peripheral arterial disease showed the highest values of sclerostin compared to those without this event (77.01 ± 26.57 *vs* 53.33 ± 23.65 pmol/L, *p* = 0.012) (Fig 2A). Moreover, patients with abnormal subrogated markers of CVD had increased levels of serum sclerostin compared to those without them: carotid plaque (69.81 ± 27.26 *vs* 44.69 ± 19.61 pmol/L, *p*<0.0001); pIMT (55.84 ± 26.00 *vs* 44.64 ± 19.25 pmol/L, *p* = 0.005); and aortic calcifications (61.92 ± 26.73 *vs* 45.75 ± 20.84 pmol/L, *p* = 0.001) (Fig 2B). Participants with hypertension had increased sclerostin levels compared to those without this condition (52.25 ± 24.26 *vs* 41.97 ± 16.98 pmol/L, *p* = 0.012).

Finally, T2D patients showed increased serum sclerostin levels compared to non-diabetic individuals (53.61 ± 24.96 *vs* 41.99 ± 16.60 pmol/L, *p* = 0.0032). When participants were further divided according to sex, we found that the serum sclerostin levels were significantly higher in men than women in the entire cohort (57.10 ± 25.31 *vs* 40.28 ± 15.35 pmol/L, *p*<0.0001).

We did not find significant differences in the sclerostin levels during comparison of participants with an eGFR above and below 60 ml/min/1.73 m^2^ (49.85 ± 19.94 *vs* 48.58 ± 22.78 pmol/L, *p* = 0.859).

The bivariate analysis showed a positive association between sclerostin and age (r = 0.2511, p = 0.004), waist circumference (r = 0.2981, *p* = 0.001), systolic blood pressure (r = 0.1756, *p* = 0.04), duration of the diabetes (r = 0.3356, *p* = 0.03), carotid IMT (r = 0.4069, *p*≤0.001) and HbA1c (r = 0.3320, *p*≤0.001). Moreover, low-density lipoprotein and high-density lipoprotein levels as well as eGFR were inversely related to sclerostin levels (r = -0.1919, *p* = 0.03; r = -0.2029, *p* = 0.02 and r = -0.232, *p* = 0.008 respectively). In terms of the relationship between sclerostin and medication (statins, anti-hypertensives, oral hypoglycaemic agents and insulin) we only found a direct relationship between sclerostin and statins (r = 0.188, *p* = 0.032).

Considering the variables that generated significant differences in the sclerostin levels and those that were associated with sclerostin in the previously performed bivariate analysis, we used a multiple linear regression analysis including them as independent variables, and sclerostin as a dependent variable. The results showed that only sex was independently associated with sclerostin levels (B = 21.144 [95% confidence interval (CI) 3.737–38.551] *p*<0.018), suggesting that men might have increased serum levels of sclerostin. When we divided the entire cohort according to the presence of T2D, we found that the association between sex and sclerostin levels was stronger in the diabetes group compared to the group without diabetes (B = 17.17 [95% CI 6.40–27.93] *p* = 0.002 *vs* B = 9.80 [95% CI 1.36–18.24] *p* = 0.024).

### Sclerostin and cardiovascular mortality

A model of logistic regression was performed to assess the variables related to mortality due to cardiovascular causes. Thus, the independent variables included in the multiple logistic regression model were, apart from sclerostin, those variables biologically linked to mortality risk (age, presence and duration of diabetes, sex, prevalent CVD, pIMT, tobacco use, hypertension and eGFR). We found that only sclerostin levels were independently associated with cardiovascular mortality (OR 1.402 [95% CI 1.019–1.927] *p* = 0.038).

To test the possible influence of the current medication used by participants on cardiovascular mortality, the same analysis was carried out, including the different drugs used as independent variables (statins, anti-hypertensives, oral hypoglycaemic agents and insulin). Similarly, we found that sclerostin remains the only variable that showed a direct impact on cardiovascular mortality independently of the other variables (OR 1.435 [95% CI 1.007–2.044] *p* = 0.046).

To assess the usefulness of sclerostin as a marker for high cardiovascular mortality risk, a ROC curve analysis was performed. The AUC, including only sclerostin, was 0.849.

**Fig 3:**
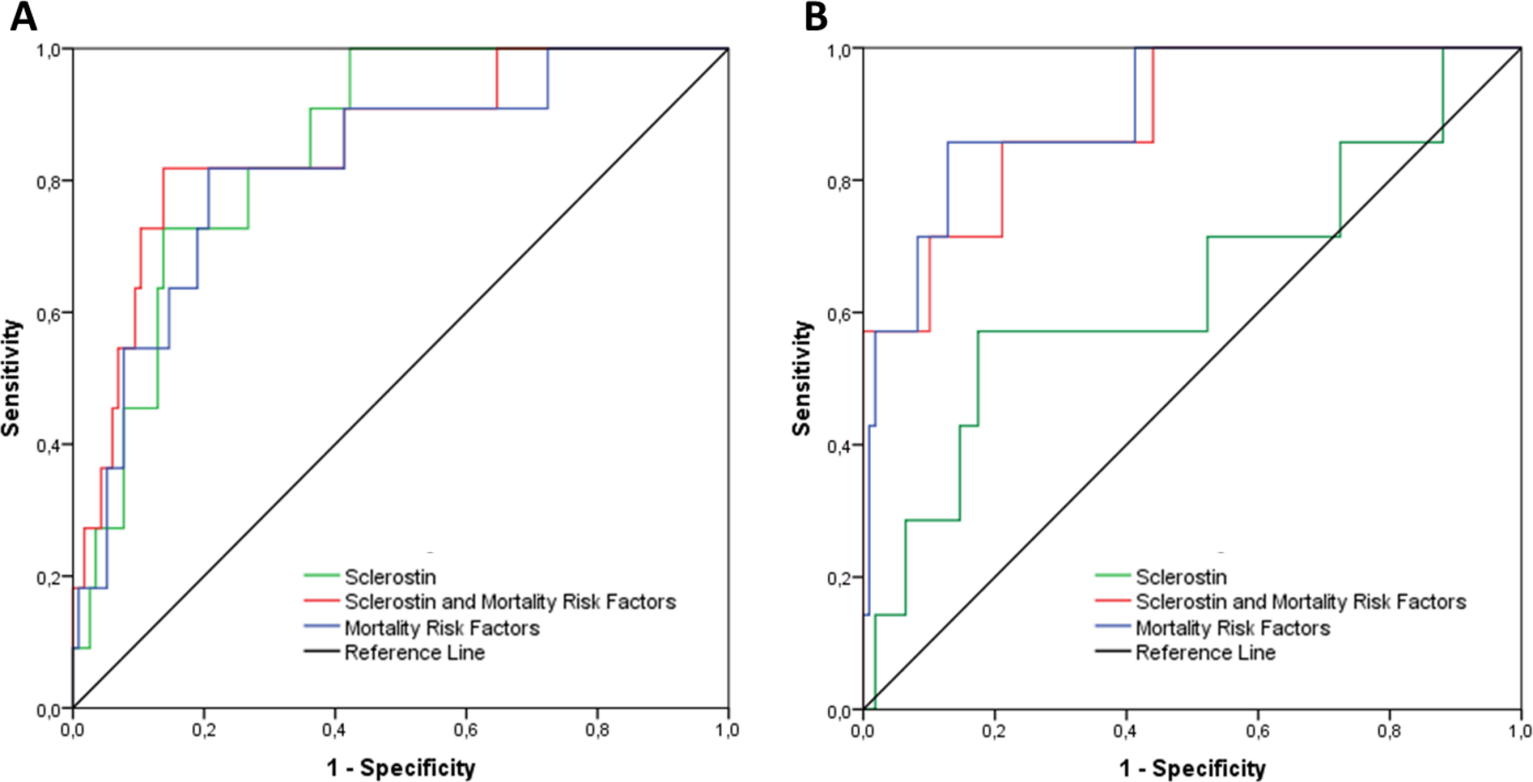
ROC curves testing the usefulness of sclerostin levels as predictors of mortality using a multiple logistic regression model. (A) ROC curve for cardiovascular mortality; (B) ROC curve for non-cardiovascular mortality. Sclerostin (A: AUC 0.849, 95% CI = 0.758–0.940, p<0.001; B: AUC 0.773, 95% CI = 0.651–0.896, p<0.001). Sclerostin and mortality risk factors (age, presence and duration of diabetes, sex, prevalent CVD, pIMT, tobacco use, hypertension and eGFR) (A: AUC 0.856, 95% CI = 0.736–0.975, p<0.001; B: AUC 0.869, 95% CI = 0.788–0.949, p<0.001). Mortality risk factors (A: AUC 0.823, 95% CI = 0.694–0.952, p<0.001; B: AUC 0.828, 95% CI = 0.740–0.915, p<0.001). ROC: receiver operating characteristic curve; AUC: area under the curve; CVD: cardiovascular disease; pIMT: pathological intima media thickness; eGFR: estimated glomerular filtration rate; CI: confidence interval.

Two additional models were assessed, including the same classical cardiovascular risk factors as in the previously conducted logistic regression model, with and without sclerostin. The AUCs of these models were 0.856 and 0.823, respectively (Fig 3A).

### Sclerostin and non-cardiovascular mortality

As in the previously performed analysis, we evaluated the usefulness of sclerostin levels as predictors of mortality due to a non-cardiovascular cause using a multiple logistic regression model. Including the same variables used in the previously performed model, we found that serum sclerostin levels were not significantly associated with non-cardiovascular mortality (*p* = 0.346).

The AUC, including only sclerostin levels as predictors of non-cardiovascular mortality, was 0.638, indicating the poor predictive power of sclerostin for non-cardiovascular mortality. Additional models, including the classical mortality risk factors with and without sclerostin, were also evaluated, showing AUCs of 0.893 and 0.906, respectively (Fig 3B).

### Sclerostin and mortality in T2D patients

To corroborate our previous results, a sub-analysis was performed in the group of T2D patients. The independent variables included in the multiple logistic regression model were the same that in the previous analysis. We found that only sclerostin levels were independently associated with cardiovascular mortality in T2D group (OR 1.495 [95% CI 1.011–2.212] p = 0.044). No significative association was found between sclerostin and non-cardiovascular mortality in T2D patients.

### Competing risk analysis

To assess the risk of cardiovascular and non-cardiovascular mortality during the follow-up period, we used the Fine and Gray model for the competing risk analysis.

**Fig 4:**
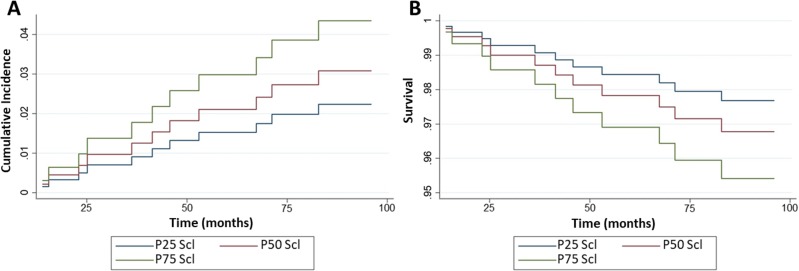
Cumulative incidence function and survival curve according to the quartiles of sclerostin for cardiovascular mortality. (A) Cumulative incidence of mortality from the Fine and Gray model and (B) Kaplan-Meier curve of the survival analysis from the proportional hazard model. The values of sclerostin are expressed as pmol/L x 10 units.

The sub-distribution hazard ratio (SHR) of sclerostin for cardiovascular mortality, adjusting for exposure time (dependent variable) and taking into account, in addition to sclerostin, the classical risk factors for mortality (age, presence and duration of diabetes, sex, prevalent CVD, pIMT, tobacco use, hypertension and eGFR), as independent variables, was 1.31 [95% CI 1.085–1.581] *p* = 0.005. In this case, for each 10 pmol/L increase in the sclerostin levels, the mortality due to cardiovascular causes increased by 31%, considering the exposure time and increased cumulative risk of cardiovascular mortality (Fig 4A).

The same analysis was performed for non-cardiovascular mortality. In this model, the circulating sclerostin levels were not significantly related to mortality (SHR 1.292 [95% CI 0.734–2.274] *p* = 0.374). Only the duration of diabetes and prevalent CVD were independently associated with mortality due to non-cardiovascular causes, considering the exposure time (SHR 0.879 [95% CI 0.779/0.993] *p* = 0.040; SHR 18.315 [95% CI 1.265/265.189] *p* = 0.033, respectively).

### Survival analysis according to the Cox model and Kaplan-Meier test

We performed the survival analysis of our data using a Cox model and Kaplan-Meier test, including the same variables used in the Fine and Gray model. By this method, we observed the same trend as in the case of the results of the Fine and Gray model, suggesting a direct relationship only between sclerostin levels and cardiovascular mortality (Table 2). Likewise, the Kaplan-Meier curve shows that higher levels of serum sclerostin are related to a lower survival percentage for cardiovascular mortality (Fig 4B).

**Table 2:** Factors independently associated with mortality in the entire cohort, using a Cox proportional hazards model.

Cardiovascular mortality	HR	95% CI	*p*
Sclerostin levels	1.318	1.090–1.595	0.004
**Non-cardiovascular mortality**			
Sclerostin levels	1.312	0.763–2.257	0.325
Duration of diabetes	0.871	0.760–0.998	0.048
Prevalent CVD	20.903	1.549–282.066	0.022

Cox proportional hazards model of cardiovascular and non-cardiovascular mortality, adjusting the results of the multiple regression analysis, with exposure time as a dependent variable and sclerostin, age, presence and duration of diabetes, sex, prevalent CVD, pIMT, tobacco use, hypertension and eGFR as independent variables.

HR: hazard ratio; CI: confidence interval; CVD: cardiovascular disease; pIMT: pathological intima media thickness; eGFR: estimated glomerular filtration rate.

## Discussion

Our results show, for the first time, that serum sclerostin is a factor independently associated with cardiovascular mortality in a study population without end-stage renal disease (ESRD). This result suggests that sclerostin levels may acts as an independent key predictor of cardiovascular mortality in a mixed population including subjects with and without T2D. Furthermore, our results showed higher levels of sclerostin in T2D patients compared to non-diabetic participants, and in men compared to women. The association between sclerostin levels and cardiovascular mortality remained when the group of diabetic subjects was analyzed separately. Patients with prevalent CVD showed increased levels of sclerostin compared to those without CVD, independently of the presence of T2D, suggesting the possible role of sclerostin in the atherosclerotic process.

Sclerostin is as regulatory molecule in bone metabolism that inhibits bone formation, mainly through binding to the Wnt co-receptor low-density lipoprotein receptor related-protein 5/6. Sclerostin has been shown to be involved in different skeletal pathologies. However, recently conducted studies have shown that sclerostin also may have several extraskeletal functions. Some studies have linked increased levels of circulating sclerostin with vascular calcification processes at the vessels [9,17]. In our previously conducted cross-sectional study, we showed increased levels of serum sclerostin in T2D patients with cardiovascular events compared to those without vascular complications. These increased levels could be explained by the expression of sclerostin in the VSMCs, under calcifying conditions [10]. According to this hypothesis, the increased sclerostin levels observed in the CVD group could be attributed to a worse cellular environment with higher levels of hyperglycaemia, insulin resistance and other cardiovascular risk factors that trigger endothelial damage thus promoting vascular calcification [28,29]. However, there are also some studies that point in the opposite direction in obese subjects without T2D, showing an inverse relationship between serum levels of sclerostin and subclinical carotid atherosclerosis as well as absence of relationship between sclerostin and parameters of cardiovascular risk. In addition, an inverse and independent association between elevated blood pressure and circulating sclerostin level was observed [30,31].

Although the majority of the previous studies pointed to an increase in the sclerostin levels in patients with CVD, the precise role of this elevation is yet to be determined. The fact that sclerostin acts as an inhibitor of bone formation suggests that increased serum levels of this protein, in patients who develop atherosclerotic processes, could be a defensive mechanism to block the Wnt canonical pathway and to reduce further progression of the calcification process. Some studies have shown an upregulation of the antagonists of the Wnt signalling pathway in patients with vascular complications [32–35]. Interestingly, a recently conducted study which evaluated romosozumab, a novel monoclonal anti-sclerostin antibody that promotes bone formation by blocking sclerostin, revealed a rapid increase in the bone mineral density, leading to a lower risk of fractures, in a large population of postmenopausal women. Nevertheless, an increased percentage of adverse cardiovascular events were reported in the group treated with romosozumab, suggesting that sclerostin inhibition could be related to cardiovascular risk [36,37]. In contrast, some studies suggested the anti-inflammatory effects of the Wnt pathway and negative effects of the antagonist of Wnt signalling over vascular lesions [38–40].

Regardless of its function at the vascular level, the evidence indicates an association between increased levels of sclerostin with vascular disorders. The increased sclerostin levels in patients with CVD make this molecule very useful to predict the risk of mortality. However, few studies have focused on the role of sclerostin as a predictor of mortality, and no data are reported in T2D patients and non-diabetic individuals. In keeping with this, our results show that high serum sclerostin levels are associated with an increased risk of mortality due to cardiovascular causes, independently of classical risk factors.

We observed similar results using the classical Cox method and Fine and Gray analysis. Our results show that sclerostin has great predictive power for cardiovascular mortality, and that it is capable of obtaining by itself an AUC higher than that obtained by including the classically established risk factors for death. Thus, the consideration of serum sclerostin levels, in addition to the risk factors, offers a trusted model for the prediction of cardiovascular mortality. This result suggests that the measurement of circulating sclerostin levels in clinical practice could be a strategy for a better estimation of cardiovascular mortality risk.

It is important to note that the predictive role of sclerostin is only applicable in the case of cardiovascular mortality and not for non-cardiovascular mortality. Some studies have reported an association between higher levels of serum sclerostin and all-cause mortality. However, this association could be attributed to the fact that CVD constitutes the major cause of death in more than half of the study subjects [20,21]. We suggest that the relationship between sclerostin and all-cause mortality is due to the weight given by sclerostin in the prediction of cardiovascular mortality.

However, it would probably not be observed if cardiovascular mortality was excluded from the causes of death, as we have observed. Similarly with our findings, Kanbay *et al*. [19] found that higher levels of sclerostin were related to an increased risk for cardiovascular events, but not all-cause mortality.

Contrary to our results, Viaene *et al*. [41] found a relationship between higher sclerostin serum levels and better survival, in ESRD patients. These conflicting results could be explained by the differences in the study populations, since individuals with an eGFR below 45 mL/min/1.73m^2^ were excluded from our study. Moreover, our multivariate analysis was adjusted for the presence of the main mortality risk factors, while the aforementioned studies adjusted only for age and sex. In addition, they used the Kaplan–Meier method to estimate cumulative incidence of mortality. Therefore, they have not considered the different events as competitive, which may lead to an overestimation of the incidence of the primary endpoint. Although the Kaplan-Meier analysis is the most conventionally used method for the estimation of time-to-death, there is growing evidence supporting a different approach that considers competing risk events [20] such as Fine and Gray model that allows for a more accurate estimation of mortality risk [25].

Our study has some limitations. First, our study population included only Caucasian individuals from a specific area, so the results cannot be extrapolated to other populations. Second, our sample size was small, owing to the strict inclusion criteria to prevent potential confounders. Finally, we did not determine sclerostin levels at the end of the follow-up period, which could provide valuable information in confirming our results. However, our study has important strengths, such as its long follow-up period (7 years) during which time cardiovascular events and deaths were assessed. In addition, our study included an exhaustive evaluation of the biochemical and clinical parameters of cardiovascular risk, integrating all the variables that could influence the study outcomes. Moreover, we conducted rigorous statistical analyses, including a competing risk analysis and the use of exposure time as an adjustment variable, in order to obtain reliable results.

## Conclusions

In summary, we demonstrated that high circulating sclerostin levels are related to mortality due to cardiovascular causes in patients with and without T2D, after adjusting for confounding variables. This finding could provide valuable information for the prediction of the risk of CVD events and the related mortality. The measurement of serum sclerostin levels in clinical practice could be a novel strategy to establish early clinical interventions in high-risk individuals. However, further studies are needed to confirm these results and to corroborate the role of sclerostin as a potential biomarker.
